# Impact of an intersectoral universal workplace intervention on health related quality of life and wellbeing in a pragmatic cluster randomised trial

**DOI:** 10.1038/s41598-025-12221-1

**Published:** 2025-07-29

**Authors:** Christoffer Lilja Terjesen, Anje Christina Höper, Erlend Hoftun Farbu, Jan Abel Olsen, Nils Fleten

**Affiliations:** 1 University Hospital of North Norway, Hansine Hansens veg 67, P.O. box 6, 9038 Tromsø, Norway; 2 Department of Community Medicine, UiT The Arctic University of Norway, Tromsø, Norway; 3 The Norwegian Labour and Welfare Administration Troms and Finnmark, Tromsø, Norway

**Keywords:** EQ-5D, Health promotion, HRQoL, Mental health, Musculoskeletal complaint, Occupational health literacy, Subjective well-being, Occupational health, Quality of life, Health policy

## Abstract

The intersectoral workplace intervention “health in work” (HIW), developed by the Norwegian healthcare service and labour and welfare administration, targets common musculoskeletal and mental health conditions by addressing both health and work environment factors. This study assessed the effectiveness of HIW on workers’ health-related quality of life (HRQoL) and subjective wellbeing (SWB) compared to standard inclusive work measures (IWM). A pragmatic cluster randomised controlled trial including 97 workplaces, randomized to either the HIW or IWM intervention over 12 months. HRQoL was measured using the EQ-5D-5L and the EQ-VAS, and SWB by using the satisfaction with life scale and a question on meaningful life. Measurements were taken at baseline, post-intervention period, and at a 12-month follow-up. EQ-5D-5L data were analysed using mixed-effects generalized linear models. No statistically significant difference-in-difference in HRQoL or SWB were found between the HIW and IWM groups at any time point. Participants in both groups reported high baseline levels of HRQoL and SWB. Although HIW did not yield significant improvements or detriments in HRQoL or SWB, this study contributes to addressing the knowledge gap regarding intersectoral collaboration in enhancing work and health. Further research is needed to assess broader outcomes such as healthcare utilisation and sick leave.

Trial registration: The trial was prospectively registered with ClinicalTrials.gov on June 24, 2019, under the identifier NCT04000035.

## Introduction

Back pain, anxiety, and depression are among the most prevalent health complaints and account for the majority of all sick leave in the organisation for economic co-operation and development (OECD) countries^1^. These conditions have far-reaching consequences for individuals, workplaces, and society, including reduced health-related quality of life (HRQoL), productivity losses, and increased healthcare utilisation^2^. Despite various efforts to prevent these health complaints, the effectiveness of such initiatives remains unclear^3,4^. Given these limitations, shifting focus towards improving individuals’ ability to manage these conditions, rather than solely attempting to prevent them, appears to be a promising approach. Indeed, universal workplace interventions that provide evidence-based health information have demonstrated positive effects on work participation and health beliefs among employees^5–7^.

Emerging evidence suggests that the psychosocial work environment plays a crucial role in employees’ overall health^8,9^. This underscores the need for a stronger collaboration between the healthcare sector and labour market stakeholders. In Norway, a national agreement was introduced in 2006 between labour unions, employer organizations, and the government to prevent and reduce sick leave while promoting work inclusion^10^. This agreement, which has been continuously renewed, led to the Norwegian Labour and Welfare Administration (Nav) established the Nav Inclusive Working Life Centres (NWC). These centres offer a variety of interventions aimed at improving the work environment and reducing absenteeism. However, the range and standardization of these NWC offered interventions seem to vary. Collectively these interventions are referred to as Inclusive Work Measures (IWM) in this paper.

To address the challenge of Norway’s high level of sick leave, a new workplace intervention, health in work (HIW), was developed in 2016. This intersectoral collaboration between tertiary healthcare services and the NWC is intended to be implemented on a national scale. HIW is specifically designed to improve employee health and well-being while reducing sick leave by integrating evidence-based health information with workplace-specific psychosocial factors. HIW consists of three structured sessions for all employees and managers at the workplace, each combining health education with facilitated group work tasks. Sessions are implemented over a 12-month period. The intervention aims to improve employees’ and employers understanding of musculoskeletal and mental health complaints. It focuses on back pain, stress, and depression, while also emphasizing the role of the workplace conditions in shaping these outcomes.

Unlike earlier mono-sectoral interventions that primarily focused on health education^5–7^, HIW also adopts a broader, intersectoral approach by incorporating strategies for managing these conditions both individually and within the organizational context. For example, during the sessions, HIW-personnel, together with employees and employers, explore the workplace’s possibilities for temporary accommodations and discuss how to support a colleague with common health complaints in the workplace. To reinforce the integration of knowledge into workplace practices, participants are to engage in structured tasks between sessions, with optional facilitation provided by NWC staff to support implementation. By targeting both individual coping mechanisms and workplace-level adaptations, HIW functions as a complex universal workplace intervention aimed at enhancing both personal and organizational health literacy, ultimately fostering improved coping strategies and reducing sick leave. For further details on the HIW and IWM interventions see the trial protocol paper^11^.

Despite the increasing recognition of workplace interventions as a strategy for improving employee health and reducing sick leave, there remains a significant knowledge gap regarding the effectiveness of complex universal workplace interventions^12–14^. To our knowledge, this is the first study to assess the effectiveness of HIW on workers’ health and wellbeing. We hypothesize that the HIW intervention will lead to improvements in health-related quality of life (HRQoL) and subjective well-being (SWB) compared to business-as-usual, represented by the IWM interventions offered by NWC. The study will evaluate the impact of HIW on these outcomes accordingly. The concepts of quality of life and subjective well-being lack universally agreed-upon definitions despite extensive efforts^15,16^. Consequently, various measurement instruments exist for assessing these constructs^17,18^. This study conceptualizes HRQoL as a multidimensional construct and employs measurement instruments that include dimensions relevant to workplace interventions, such as work participation, pain/discomfort, and anxiety/depression. Subjective well-being is in this study considered as a psychological state encompassing life satisfaction, emotional well-being, and a sense of meaning and purpose. Given that this study aims to inform policymakers at multiple levels, including workers, enterprises, and national health and labour authorities, we will apply generic measurement instruments that facilitate comparison with other health interventions.

Since both HIW and IWM are delivered within workplaces, a cluster-randomized design was chosen.

## Methods

### Trial design

To explore any changes in individual participants’ HRQoL and SWB attributable to the HIW intervention, a pragmatic cluster randomized trial design was applied. As a comparator to the HIW intervention, the usual IWM were chosen. IWM represents the current alternatives of public intervention for workplaces, delivered solely by NWC as a part of the IA-agreement^10^. The trial design has previously been reported^11^. The trial was registered at ClinicalTrials.gov (Identifier: NCT04000035) on 27/06/2019. In short, the trial was conducted as a multicentre superiority trial with two parallel arms and a 1:1 allocation ratio. A 12-month period of intervention and a 12-month follow-up period was given both arms. One arm offering the IWM interventions and the other arm offering the new HIW intervention. IWM-intervention workplaces were also excluded from receiving HIW-interventions during the follow-up period. As both IWM and HIW interventions are aimed at all employees within a workplace, cluster-randomization was conducted at the workplace level. Individual consents were obtained, and a questionnaire was digitally forwarded by the leader through work e-mail addresses before randomization (q1), through self-determined e-mail addresses after the intervention period of 12 months(q2) and finally after a follow-up period of another 12 months (q3). The trial inclusion was conducted between June 2019 and July 2021. Due to COVID-19 pandemic-related restrictions in workplaces, some measures were taken. Interventions, including HIW, were made possible to be optionally selected as digital interventions or as a hybrid digital and in-person interventions. Furthermore, to ensure achievement of 12 months of actual intervention time, the intervention period was prolonged and the time point of sending the second questionnaire was postponed accordingly. Regardless of the intervention group, prolongations were set to a total of 18 and 24 months for workplaces included in the second half of 2020, or before the governmental imposed restrictions in March 2020, respectively. For details see Appendix Table A.

### Setting

HIW or IWM interventions were carried out at workplaces throughout Norway’s two northernmost counties of Troms and Finnmark, a sparsely populated area of approximately 75.000 km^2^ and a population of 243.000 people. Interventions were conducted in geographically scattered work environments and in different types of industries. Involved personnel from NWC and the tertiary health services had varying experience and were situated at different locations throughout the counties. Personnel from the health services were employed at the University Hospital North Norway in Harstad and Tromsø, at the Rehabilitation Center of Finnmark in Alta and at the Finnmark Hospital Trust in Kirkenes. The NWC Troms and Finnmark personnel were based in local offices (municipalities of Vadsø, Hammerfest, Alta, Lakselv, Nordreisa, Målselv, Tromsø, Finnsnes and Harstad).

### Recruitment of workplaces and individual participants

Workplaces were invited to enrol in this trial by NWC personnel as a part of their regular outreach activities. In this context, a workplace was defined as a natural working environment where employees routinely collaborate and share responsibilities, regardless of formal organizational structures. Eligibility required workplaces to demonstrate motivation to improve their work environment, as assessed by NWC personnel in dialogue with workplace leaders. Additionally, workplaces needed at least eight employees and available sick-leave data (both self- and physician-certified) for the two years before allocation. Inclusion criteria for individual participants were: *i*) proficient in Norwegian, *ii*) aged 18–70, and *iii*) employed ≥ 20% in the participating workplaces. Individuals were invited by written information from the research group, and workplaces were given the opportunity to get a brief introductory presentation from a member of the research group.

### Sample size and blinding

Sample size calculation was originally performed on measures exploring significant differences in sick leave. Based on prior studies with similar work place interventions^5,7^ we estimated 50 clusters and 240 individuals in each arm to be sufficient to reveal a clinical significant difference in changes between the two trial groups at 80% strength and a 5% significance level^11^. No blinding was adopted in this study. Randomization was conducted using concealed envelopes. Details of sample size estimation and blinding procedures can be found in the protocol paper^11^.

### Outcome collection

The primary outcome measures were HRQoL and wellbeing at individual participant level. To measure HRQoL in a manner that allows for comparison across different types of health interventions and includes both the physical, social, and psychological condition of the participant, we implemented the generic preference-based measure EQ-5D-5L in the digital questionnaires. Applying this instrument is in line with Norwegian national guidelines. Health states were converted into index values from an aggregate value set based on means of ten Western countries’ preference pattern (MN-WEPP)^19^ and treated as a continuous variable from -0.578 to 1, where 1 is the best health state. To further explore HRQoL, EQ VAS scores were analysed. The EQ-VAS is a visual analogue scale to capture respondent’s overall assessment of their health on a scale from 0 (worst health imaginable) to 100 (best health imaginable). Wellbeing was measured by the first three items of the satisfaction with life scale (SWLS-3)^20,21^. Each item is rated along a 7-point scale, providing a sum score ranging from 3 representing the lowest life satisfaction up to 21 indicating highest life satisfaction. In addition, we included a global question on life meaningfulness^22^, rated on 0–10 point scale, where 0 is not at all meaningful and 10 is completely meaningful^20^. These wellbeing measures are also in line with national recommendations^23^.

### Analyses

Descriptive analysis was performed to assess baseline characteristics in trial groups to detect imbalances in need of adjustment. Baseline descriptive statistics were estimated using Chi-squared test for categorical variables and independent t-test for continuous variables. Continuous variables normally distributed were described using mean and standard deviation (SD). In case of skewed distribution, median and quartiles were applied, and categorical variables are presented as counts and percentages. For all outcomes, descriptive- and inferential statistics were performed by intention-to-treat principles and difference-in-difference analyses to address any differences between the trial groups. Additionally, inference statistics on primary outcome data at individual level was analysed using mixed effects generalized linear models (MEGLM) to account for any baseline differences between the groups, clusters- and within-subject correlation. First, EQ-5D-5L data was assigned index values based on the MN-WEPP value set. Second, we fitted a MEGLM using a gamma distribution. Third, we estimated marginal means and inverted the estimated means back to the original scale for readability. A significance levels of *α* < 0.05 was applied. Analyses were produced using STATA statistical software version 18 (StataCorp, College Station, Texas). This study is reported in accordance with the Consolidated Standards of Reporting Trials (CONSORT) guidelines from 2010, including the extension for cluster randomised trials^24^.

## Results

**Fig. 1:**
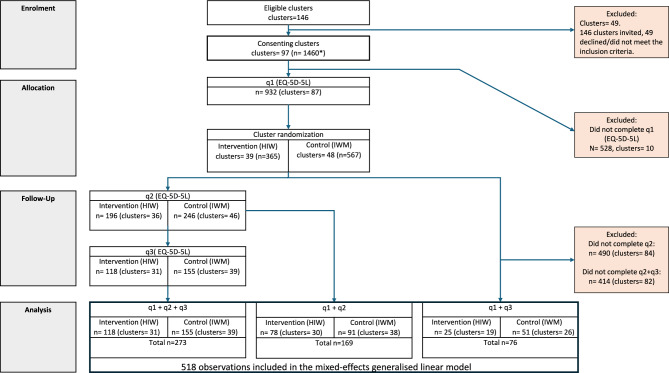
Participant flow diagram. Flowchart showing participants response rate to EQ-5D-5L questionnaires and the distribution between the intervention group and the control group. *Among the 97 included clusters, 1460 individuals consented to participate. However, precise data on the total number of eligible participants within these clusters were not available to the study group. This limitation arose because some clusters lacked access to accurate records of their total workforce, partly due to the cluster construct of the workplace disregarding formal organisational structures. 518 unique participants answered EQ-5D-5L in q1 and q2 and/or q3 and was included in the mixed-effects general linear model. *q1* = *questionnaire at baseline. q2* = *questionnaire at post-intervention period. q3* = *questionnaire at 12-month follow-up. n* number of responding participants. *HIW* health in work. *IWM* inclusive workplace measures. *SD* standard deviation.

A total of 97 workplaces with 1460 consenting participants were randomized. 45 workplaces were allocated to the intervention group and 52 workplaces to the control group. The question on EQ-5D-5L in the first questionnaire was answered by 64% of the consenting participants, respectively 365 in the intervention group and 567 in the control group. 30% of the 1460 consenting participants answered the second questionnaire and 24% answered the third questionnaire. For more details refer to (Fig. 1).

Baseline descriptive statistics detected no significant differences at cluster level between the groups when comparing type of sector, type of business, geographical dispersion, location, year intervention ended and whether they were given extended time due to the COVID-19 pandemic (Appendix Table B). However, at individual level some significant differences were detected. 12% of the participating individuals in the intervention group were employed in the industry category vs 20% in the control group. Opposite, 28% were employed in the category of education in the intervention group vs 16% in the control group. 81% of the individuals were women in the intervention group vs 72% in the control group. Some age differences were detected as the intervention group had less individuals in the category age 30–39 (20 vs 24%), but more in the age category >  = 60 (13 vs 8%). Also, the geographical dispersion was significantly different with proportionally fewer participating individuals in Troms county than Finnmark county in the intervention group (61 vs 75%). The location of their workplaces showed statistically significant differences between the groups as well, showing fewer individuals in urban areas than rural in the intervention group (56 vs 76%). Primary outcome measures of HRQoL descriptive statistics showed no significant differences at baseline or at follow-up between the groups (Table 1).

**Table 1:** Descriptive statistics of EQ-5D-5L scores.

Descriptive statistics of EQ-5D-5L score (MN-WEPP weighted) based on **all answers**					
Time	n	Intervention (HIW) Median (quartile 1 – quartile 3)	n	Control (IWM) Median (quartile 1 – quartile 3)	
q1	365	0.93 (0.869–1)	567	0.93 (0.847–0.930)	
q2	255	0.94 (0.879–0.942)	327	0.94 (0.832–0.940)	
q3	203	0.94 (0.877–0.940)	274	0.90 (0.835–0.940)	

Descriptive statistics of EQ-5D-5L score (MN-WEPP weighted) based on **2 < answers**					
Time	n	Intervention (HIW) Median (quartile 1 – quartile 3)	n	Control (IWM) Median (quartile 1 – quartile 3)	
q1	221	0.93 (0.836–0.932)	297	0.93 (0.847–1)	
q2	196	0.94 (0.879–0.940)	246	0.94 (0.843–1)	
q3	143	0.94 (0.843–1)	206	0.94 (0.835–0.940)	

Descriptive statistics of **change** in EQ-5D-5L score (MN-WEPP weighted)					
Time	n	Intervention (HIW) mean change (SD)	n	Control (IWM) median mean change (SD)	p-value
q1→q2	196	−0.007 (0.099)	246	0.001 (0.115)	0.4656
q2→q3	118	0.005 (0.094)	155	0.006 (0.089)	0.9484
q1→q3	143	0.008 (0.109)	206	-0.003 (0.085)	0.2684

Descriptive statistics of weighted score results from questionnaires. No statistically significant differences in differences were detected.

*n* number of responding participants. q1 = questionnaire at baseline. q2 = questionnaire at post-intervention period. q3 = questionnaire at 12-month follow-up. *HIW* health in work. *IWM* inclusive workplace measures. *SD* standard deviation.

To provide a more detailed understanding of the distribution of responses, we examined the most frequently reported EQ-5D-5L profiles and EQ-VAS scores across participants (Appendix Table E).

**Fig. 2:**
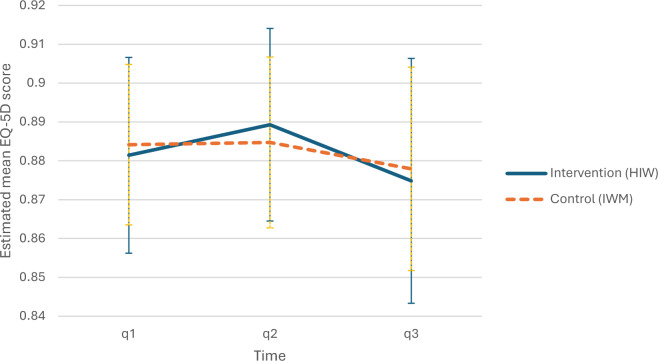
Mixed-effects generalised linear model estimated mean EQ-5D Scores. Primary outcome analysis using a multilevel mixed-effects generalised linear model with 95% confidence intervals showed no statistically differences between the two trial groups. The Y-axis represents EQ-5D score values based on the MN-WEPP value sets and the x-axis indicates the time by questionnaire 1–3. 95% Confidence Intervals are applied. q1 = questionnaire at baseline. q2 = questionnaire at post-intervention period. q3 = questionnaire at 12-month follow-up.

To address the hierarchical data structure and intra-class correlation along with some unbalanced baseline data, a MEGLM analysis was performed. This showed no significant differences between the groups. Adjusting for age, sex, geographical dispersion, location and type of business in the MEGLM, neither showed any statistically significant differences between the intervention and control group (Fig. 2).

Simple descriptive statistics from the EQ-VAS scores treated as a continuous variable neither indicates differences in differences between the two groups (Table 2).

**Table 2:** Descriptive statistics of EQ-VAS scores.

Descriptive statistics of EQ-VAS scores, based on respondents answering 2 < EQ-5D-5L questionnaires					
Time	n	Intervention (HIW) Median (quartile 1 – quartile 3)	n	Control (IWM) Median (quartile 1 – quartile 3)	
q1	215	0.80 (0.70–0.90)	293	0.80 (0.70–0.90)	
q2	191	0.80 (0.70–0.87)	242	0.80 (0.70–0.89)	
q3	141	0.78 (0.68–0.90)	202	0.80 (0.72–0.88)	

Descriptive statistics of change in EQ-VAS scores, based on respondents answering 2 < EQ-5D-5L questionnaires					
Time	n	Intervention (HIW) Mean change (SD)	n	Control (IWM) Median Mean change (SD)	p-value
q1→q2	188	−2.2606 (15.70)	241	−0.0083 (12.85)	0.1031
q2→q3	116	1.7414 (13.60)	154	−0.6104 (11.46)	0.1247
q1→q3	138	−1.9420 (17.18)	200	−0.4000 (14.20)	0.3688

Descriptive statistics of EQ-VAS scores based on respondents who answered the EQ-5D-5L questionnaire. No significant differences in change were detected in this simple difference in difference analysis. q1 = questionnaire at baseline. q2 = questionnaire at post-intervention period. q3 = questionnaire at 12-month follow-up.

*n* number of responding participants. *HIW* health in work. *IWM* inclusive workplace measures. *SD* standard deviation.

Descriptive statistics of the questionnaires related to SWB showed no difference between the two groups. The composite SWLS-score based on the three items showed no significant difference. The question on life meaningfulness showed general high levels in both the intervention and control group with no significant difference in difference change (see Appendix Table C).

Due to the COVID-19 pandemic, workplaces had the option to receive interventions either through live online sessions or a hybrid format that combined in-person participation with interactive video-based delivery. The live online or hybrid intervention format was implemented in 9 of the 45 businesses (For more details see Appendix Table D). Due to pandemic-related restrictive policies, 32% of the total workplaces were given additional intervention time, deviating from the original protocol (see Appendix Table A). This additional time was distributed quite similar between the groups with 27% of the intervention group and 37% of the control group (Appendix Table A).

## Discussion

### Effectiveness of the HIW intervention

This study aimed to evaluate the effectiveness of the HIW intervention compared to business-as-usual IWM on HRQoL and SWB. Our results suggest no significant effect of the HIW intervention on HRQoL compared to IWM, when measured by EQ-5D-5L or EQ-VAS. Similarly, on SWB, no significant differences were observed between the groups when measured by SWLS-3 or by meaningfulness. To our knowledge, there is a lack of prior research on similar interventions targeting a general working population while measuring HRQoL or SWB, making direct comparisons with existing literature challenging. Given this knowledge gap, our study contributes valuable insights into the potential effects of workplace interventions on these outcomes. We only identified one relevant study from 2013 by Odeen and colleagues^5^ who conducted a universal mono-sectoral workplace intervention educating on low-back pain. This study reported a significant reduction in sick leave but, consistent with our findings, found no significant difference in self-rated health between the intervention and control groups, as measured by a single-item five-point scale.

### Strengths of the study design

A key strength of this study is its cluster-randomized controlled trial design, which minimizes selection bias and increases the reliability of the results. The study’s broad geographical distribution of workplaces, as well as the inclusion of both public and private sectors, mitigates the risk of inter-cluster contamination and enhances the generalizability of the findings. Moreover, the heterogeneous mix of businesses and job positions, as well as varying levels of team experience delivering the interventions, further strengthens the external validity of the study. Another notable strength is that the analyses are based on an intention-to-treat approach, reflecting the real world and therefore providing a pragmatic view of how the HIW will perform in a broader population. Finally, using the generic preference-based EQ-5D-5L instrument allows for cross-sector and cross-population comparisons of health interventions, providing valuable input for policymakers in occupational health.

### Limitations of the study

This study has several limitations that must be considered when interpreting the results. The participating workplaces may not fully represent all Norwegian workplaces, since participation was based on motivation to improve or maintain the work environment. Of the 97 included clusters, a total of 1,460 individuals provided consent to participate in the study. However, exact information on the total number of eligible participants within these clusters was not available. This limitation was due to some clusters lacking access to precise workforce records, partly because the cluster design used for the workplace did not align with formal organizational structures. Another limitation is that the study encountered higher-than-expected rates of missing data from the questionnaires, which may limit the representativity of the results. Missing data is a well-documented challenge in occupational health interventions, especially in randomized controlled trials^25,26^, and high rates of non-response can affect the reliability of the findings.

### Ceiling effects in HRQoL measures

The study population exhibited a high baseline level of HRQoL, as the distribution of EQ-5D-5L values was heavily skewed towards good health. As detailed in Appendix Table E, the majority of participants reported EQ-5D-5L profiles indicating no, or only slight, health-related impairment, and a high proportion had EQ-VAS scores at the upper end of the scale. In a generally healthy group of participants, there is limited room for measurable improvement, which constrains the ability of the these instrument to detect meaningful changes^27^. To address this, future studies might consider to include additional psycho-social dimensions that may better capture changes in HRQoL and SWB, particularly in already healthy populations.

### Impact of the COVID-19 pandemic

The COVID-19 pandemic introduced challenges that may have influenced the study results, particularly due to restrictions on workplace presence, affecting intervention delivery. Since the interventions rely on shared knowledge and group-based tasks, these restrictions may have impacted feasibility. In response to Norway’s pandemic policies, NWC interventions, including HIW, were offered as digital or hybrid (in-person with video) formats. This was in line with suggested mitigation strategies to adapt to constraints imposed by pandemic responses^28^. However, only 20% of workplaces used these formats, and additional intervention time was similarly distributed between groups.

### Implications for policy and future research

Our findings suggest that incorporating the HIW-intervention into standard practice should not be expected to greatly increase HRQoL or SWB in the target population compared to delivering the business-as-usual mono-sectoral IWM from Nav. Nor should HIW have any negative enactments. Challenges surrounding measuring HRQoL and SWB in generally healthy populations could hamper the policymakers in prioritization of universal workplace health interventions and thus support the need for further methodological research. To evaluate and capture the full impact of the HIW intervention, future research should also explore other relevant outcomes. Failure to capture the whole range of effects could jeopardize rational decision-making. For HIW it would be appropriate to additionally evaluate a wider spectrum of relevant outcomes, such as healthcare utilisation and sick leave, to address multiple stakeholder interests.

## Conclusions

In a population with already high levels of health-related quality of life and subjective wellbeing, this study did not detect any improvement or deterioration in these outcomes following the implementation of the HIW workplace intervention compared to the business-as-usual IWM approach. The generally high baseline levels of HRQoL and SWB in this working population, and the generic instruments used, may have limited the ability to detect subtle improvements. Additionally, the presence of missing data suggests that the findings should be interpreted with caution. These results highlight the methodological challenges of evaluating universal, complex workplace interventions. Since HRQoL and SWB are not the only relevant outcomes of these interventions, more research is needed to adequately evaluate the HIW intervention from multiple perspectives and involved payers.

## Supplementary Information

Supplementary Information 1.

Supplementary Information 2.

## Data Availability

The datasets generated and/or analysed during the current study are not publicly available due to legal and ethical restrictions under Norwegian data protection legislation. Data are available from the corresponding author on reasonable request and subject to appropriate ethical approvals.

## Abbreviations

CONSORT: Consolidated standards of reporting trials
EQ-5D-5L: EuroQol 5-dimension scale 5-level version
EQ-VAS: EuroQol visual analogue scale
HIW: Health in work
HRQoL: Health-related quality of life
IA-agreement: Inclusive work agreement
IWM: Inclusive work measures
MEGLM: Mixed effects generalized linear models
MN-WEPP: Means of ten western countries’ preference pattern
Nav: Norwegian labour and welfare administration
NWC: Nav inclusive working life centres
OECD: Organisation for economic co-operation and development
SWB: Subjective well-being
SWLS-3: Satisfaction with life scale, 3-item version
