# Supplementary material for: Impact of an intersectoral universal workplace intervention on health related quality of life and wellbeing in a pragmatic cluster randomised trial
Source: Sci Rep. 2025 Jul 29;15:27715. doi: 10.1038/s41598-025-12221-1 (PMC12307591; doi:10.1038/s41598-025-12221-1)
Supplement: Supplementary file 2 — Supplementary Information 2. [file 41598_2025_12221_MOESM2_ESM.pdf]

Appendix Table C. Descriptive statistics on satisfaction with life (SWLS-3) (3-21 scale) and meaningful life (0-10 scale).

Descriptive statistics of satisfaction with life (3-21), based on the population that responded to the EQ-5D-5L (n=518).					
Time	n	Intervention (HIW) Median (quartile 1 – quartile 3)	n	Control (IWM) Median (quartile 1 – quartile 3)	
Q1	217	15 (13-18)	294	15 (13-18)	
Q2	193	15 (13-18)	244	15 (13-18)	
Q3	142	15 (13-17)	204	15 (13-18)	
Descriptive statistics of change in satisfaction with life (3-21)					
Time	n	Intervention (HIW) Mean change (SD)	n	Control (IWM) Median Mean change (SD)	p-value
Q1→Q2	190	-0.511 (2.708)	242	-0.045 (3.330)	0.1191
Q2→Q3	116	0.155 (2.682)	152	-0.171 (2.671)	0.3236
Q1→Q3	139	-0.331 (2.786)	202	-0.015 (3.180)	0.3005

Descriptive statistics of meaningfulness in life (0-10), based on the population that responded to the EQ-5D-5L (n=518).					
Time	n	Intervention (HIW) Median (quartile 1 – quartile 3)	n	Control (IWM) Median (quartile 1 – quartile 3)	
Q1	221	8 (7-9)	297	8 (7-9)	
Q2	196	8 (7-9)	248	8 (7-9)	
Q3	143	8 (7-9)	205	8 (7-9)	
Descriptive statistics of change in meaningfulness in life (0-10)					
Time	n	Intervention (HIW) Mean change (SD)	n	Control (IWM) Median Mean change (SD)	p-value
Q1→Q2	196	-0.168 (1.206)	248	-0.165 (1.509)	0.9816
Q2→Q3	118	-0.076 (1.385)	156	-0.058 (1.137)	0.9031
Q1→Q3	143	-0.203 (1.303)	205	-0.146 (1.393)	0.7027
